# Production of proteolytic extract by *Aspergillus oryzae *grown by solid state fermentation using canola meal as substrate

**DOI:** 10.1186/1753-6561-8-S4-P177

**Published:** 2014-10-01

**Authors:** Natália Lima de Oliveira Pascoal, Suzanne Alves Cordeiro, Gustavo Adolfo Saavedra Pinto

**Affiliations:** 1 Ceará State University, RENORBIO, Fortaleza, CE, Brazil; 2 National Research Center of Tropical Agroindustry, Embrapa, Fortaleza, CE, Brazil

## Background

Proteases are enzymes used in diversified process and industrial products, where the most important is the food sector and the detergent fabrication. The microbial obtention way is the most relevant, and the filamentous fungi are the main synthesis agents in industrial scale. [1]. The genres *Conidiobolus, Verticillium, Penicillium *e *Aspergillus *are reported as proteases producers. The specie *Aspergillus oryzae *is considerate not toxicogenic and it is a huge proteases producer, so it has an elevated industrial interest [2,3]. The literature about this theme is vast, there are countless articles using the *Aspergillus *in semi-solid fermentative processes to proteases of obtention. Nevertheless, the importance of this work was demonstrated by the use of an alternative substrate to protease synthesis, the canola meal. The canola meal it is a byproduct of the extraction process of canola oil, showing high protein content. Nowadays, the industrial sector has encouraged researches that can contribute and adding value to this residue [3]. So, in this context, this work proposed to evaluate preliminarily the performance of five lines of *Aspergillus oryzae *in relation to the production of proteases in semi-solid fermentation using the canola meal as a substrate inductor.

## Methods

Five NRRL strains of *Aspergillus oryzae *(designated 2220, 1911, 5590, 694 e 2217) were used in these experiments. For the solid state fermentation was used a medium of canola meal at proportion of 100g of bran to 40mL of water. The experiments was done in Erlenmeyers of 500mL, each flask contained 40g of medium, previously autoclaved. The initial inoculum was of 10^7 ^spores.g^-1 ^of fermentation medium. The fermentations were performed in statics conditions under temperature of 20°C during 96 hours. The proteolytic activity was determined each 24 hours using azocasein as substrate and trichloroacetic acid as precipitating agent. A unit of proteolytic activity was defined as a quantity of enzyme that produces a difference of 0.01 of absorbance per minute of reaction between the reactional blank and the samples in the experimentals conditions. The results were expressed in U.g^-1 ^of fermentation medium [5]. The statistical analysis, through the Scoot-Knott test was performed.

## Results and conclusions

Within 48 hours of fermentation, all extracts showed proteolytic activity, indicating that all strains were able to synthetise proteases. The highest levels of activity were reach at 72 hours of fermentation and no difference was observed after this period. It was observed that *A. oryzae *NRRL 2217 and NRRL 2220 were protease best producers. The proteases production profile of the canola bran was similar to that demonstrated by Thanapimmetha [4], with *Jatropha curcas *residue medium. This study shows the viability of using the canola meal to the synthesis of proteases by differents strains of *A. oryzae*.

## References

[B1] Hernández-Martínez R Gutiérrez-Sánchez G Bergmann CW Loera-Corral L Rojo-Domínguez A Huerta-Ochoa S Regalado-González C Prado-Barragán LA Purification and characterization of a thermodynamic stable serine protease from Aspergillus fumigates Process Biochemistry 2011 46 2001 2006 doi.org/10.1016/j.procbio.2011.07.013 10.1016/j.procbio.2011.07.013

[B2] Ward OP Industrial Biotechnology and Commodity Products.Proteases In Comprehensive Biotechnology 2011 3 2nd University of Waterloo, Canada 571 582 doi:10.1016/B978-0-08-088504-9.00222-1

[B3] Aluko RE McIntosh T Limited enzymatic proteolysis increases the level of incorporation of canola proteins into mayonnaise Innovative Food Science & Emerging Technologies 2005 6 2 195 202 doi.org/10.1016/j.ifset.2004.11.003 10.1016/j.ifset.2004.11.003

[B4] Thanapimmetha A Luadsongkram A Titapiwatanakun B Srinophakun P Value added waste of Jatropha curcas residue: Optimization of protease production in solid state fermentation by Taguchi DOE methodology Industrial Crops and Products 2012 37 1 5 10.1016/j.indcrop.2011.11.003

[B5] Charney J Tomarelli RM A colorimetric method for the determination of the proteolytic activity of duodenal juice Journal of Biological Chemistry 1947 170 23 501 505 20272088

